# A comprehensive technique for artificial hybridization in Chickpea (*Cicer arietinum*)

**DOI:** 10.1186/s13007-017-0202-6

**Published:** 2017-06-21

**Authors:** Shweta Kalve, Million Tadege

**Affiliations:** Department of Plant and Soil Sciences, Institute for Agriculture Biosciences, Oklahoma State University, 3210 Sam Noble Parkway, Ardmore, OK 73401 USA

**Keywords:** Chickpea, Artificial hybridization, Hand pollination, Flower stage, Genetic crossing

## Abstract

**Background:**

Two crossing techniques for hybridization of chickpea have been reported and include pollination after emasculation and pollination without emasculation. Success of crossing with emasculation varied from 5 to 17%; while the success rate varied from 20 to 50% by pollination without emasculation. The important reason for the low success rate of the two procedures could be lack of detailed information on the flowering stages chosen for crossing together with the environment where plants grow.

**Results:**

We describe a comprehensive method for chickpea crossing where two genotypes, ICCV96029 as female and PI503023 as male parent were used. Leaf shape and seed size were used as morphological markers to select hybrids. For crossing, incision was made along the central line of the keel petal for the removal of anthers and to expose the stigma for placement of pollen from donor parent on its surface. After pollination, style was inserted back gently inside the keel petal and covered by wing petals and standard petals to make a natural sac which prevents drying of internal organs. Alternatively, if the conditions are favorable there is no need to protect the pollinated flower and therefore petal removal method for cross-pollination can be used. Our method showed around 78% crossing success rate which is much higher than the previous results.

**Conclusions:**

We have shown that the crossing by keel petal incision or petal removal is an effective approach which significantly increases the crossing success rate. Furthermore, our detailed method shows that the flowering stage, selection of parents and temperature play crucial roles in crossing success.

**Electronic supplementary material:**

The online version of this article (doi:10.1186/s13007-017-0202-6) contains supplementary material, which is available to authorized users.

## Background

Chickpea (*Cicer arietinum*) is the second largest cultivated food legume crop in the world [1]. It is an excellent source of energy and nutrients having high quality protein, with a wide range of essential amino acids [2]. Its use both as human food and animal feed, coupled with its ability to fix atmospheric nitrogen makes it a very important crop. There are two distinct types of chickpea genotypes; desi and kabuli, based primarily on seed size, shape, and color. The desi type is mostly grown in Asia and Africa while the kabuli type is commonly found in Mediterranean region and also widely grown in North America, particularly in Mexico and US [1, 2].

In the past few years many advances in chickpea genomics have provided more opportunities to explore unique chickpea genomic characteristics and evaluation of their biological significance, including advances in draft genome and transcriptome sequencing [3, 4]. However, conventional breeding methods are still most frequently used to develop new genotypes in this crop. Chickpea is predominantly a self-pollinating species and due to its small flowers, crossing is difficult and tedious.

Two methods have been reported for genetic crossing in chickpea: artificial hybridization with and without emasculation [5, 8–12, 14] with very low success rate. To our knowledge comprehensive method for chickpea crossing by emasculation is not available. Nevertheless, few reports have shown various ways to improve crossing techniques. Tullu and van Rheenen [5] showed that field environment is more favorable for crossing chickpea than green house. Moreover, they have reported that time of emasculation and pollination has no significant effect on crossing success. In contrast, emasculation followed by pollination was found to be more effective in some parts of the world while evening emasculation and next day pollination was found to have better results in other parts [12]. Other factors that were reported to affect the crossing efficiency were temperature and humidity [5, 6]. Selection of parents for crossing has also been found crucial for successful hybridization [7]. On the other hand, crossing devoid of emasculation was found as a second option for chickpea crossing [8–11]. For this method to succeed, identification of flower stage is very important so that the artificial pollination can be done before its pollen grains are shed naturally. The success rates in previously described studies are less ranging from 5 to 50%. The important reason for low crossing success in both the methods, in addition to environmental factors, could be the lack of comprehensive information about flowering stages of chickpea chosen for crossing.

Our study provides an easy and efficient technique for chickpea crossing by keel petal incision or petal removal (Additional file 1: Video S1; https://youtu.be/ZTgDUcLGc_o), where detailed knowledge about the selection of flower stages has been described.

## Results and discussion

### Selection of parents and favorable conditions for artificial hybridization

**Fig. 1:**
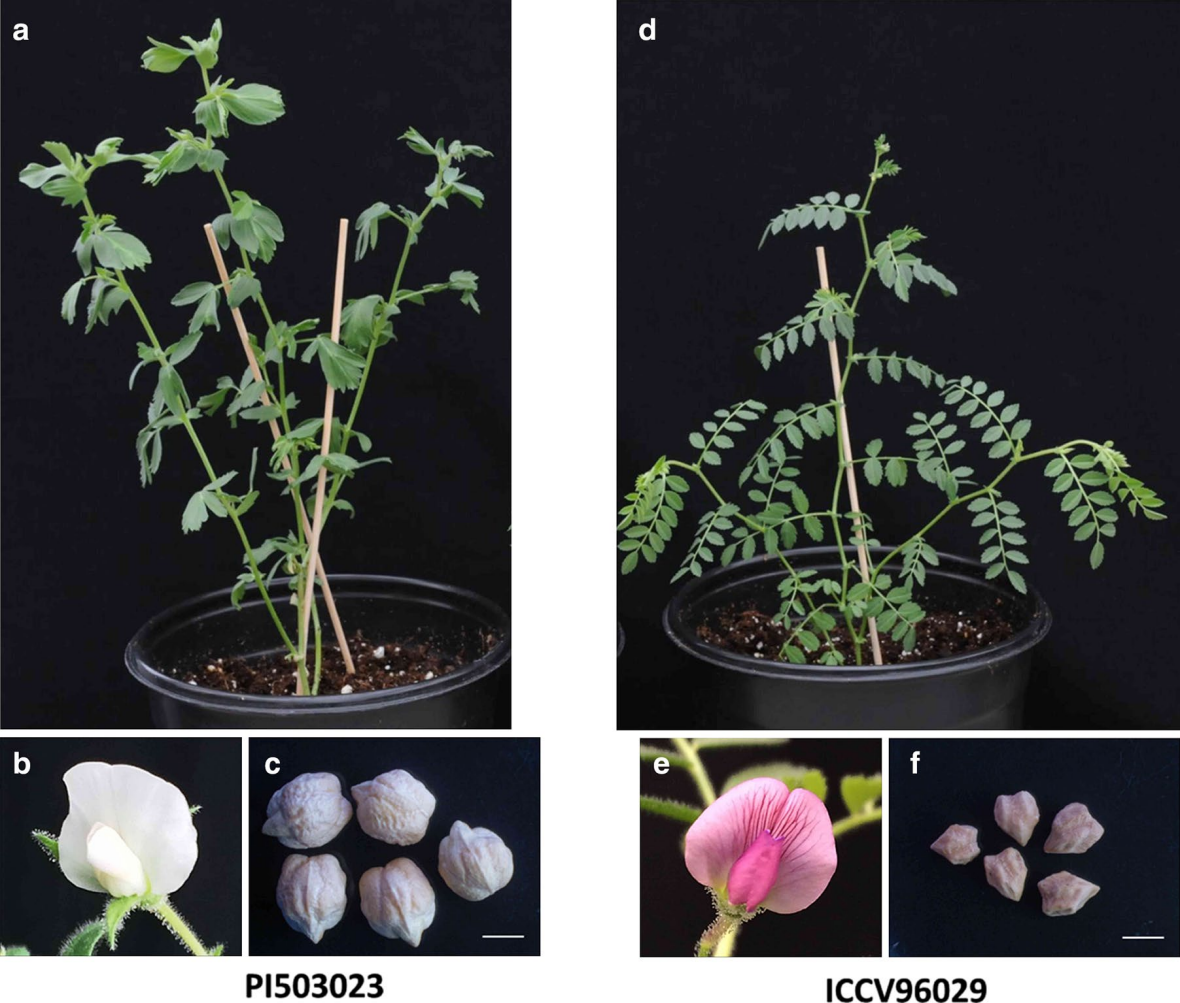
Chickpea genotypes used for crossing. **a**–**c** Simple leaf, white flower and kabuli seeds of male parent (PI503023). **d**–**f** Compound leaf,* purple* flower and desi seeds of female parent (ICCV96029). *Bars* 5 mm

Parental selection is the first step for genetic crossing. In present study we have used two chickpea genotypes having significant differences in leaf shape, seed size, flower color and flowering time. ICCV96029 is an early flowering desi cultivar that has compound leaf and purple flower while PI503023 is a late flowering kabuli cultivar and has simple leaf and white flower (Fig. 1). ICCV96029 was selected as a female parent and PI503023 as male parent. Mainly, leaf shape and seed size were used as morphological markers to select hybrids as our main target is to study leaf and seed genetics. It has been reported that crossing success may be influenced by the parental identity and the environment in which plants are growing [13]. The female parent plays a crucial role in determining the crossing success between both the parents. Therefore, it is essential to select the precise male and female parent for crossing. It was reported that for better crossing in chickpea, parent with small seed size should be used as female parent [7]. Moreover, female flower with anthocyanin pigmentation is better than the one without pigmentation which often scheduled for natural flower drop [12]. Therefore, for correct parental selection we used both the cultivars as male and female parent. We observed that when PI503023 was selected as female parent, the survival of the crosses was reduced by around 70–80% and therefore ICCV96029 was used as female parent. Consistent with the previous findings, our study shows that the small seeded female parent having purple flower increases the crossing success.

We have observed that seed set was around 20–30% higher when emasculation followed by artificial pollination was performed in relatively cooler environment such as in the morning between 08:00 and 10:00 h or 17:00 and 18:00 h in the evening. Additionally, we found a high success rate at optimum temperature between 22 and 26 °C whereas increase in temperature to more than 30 °C reduced survival by approximately 40%. Similar to our studies the negative effect of high temperature was also reported to affect seed setting in chickpea [14].

### Developmental stages of chickpea flower and precise flower stage selection for crossing

**Fig. 2:**
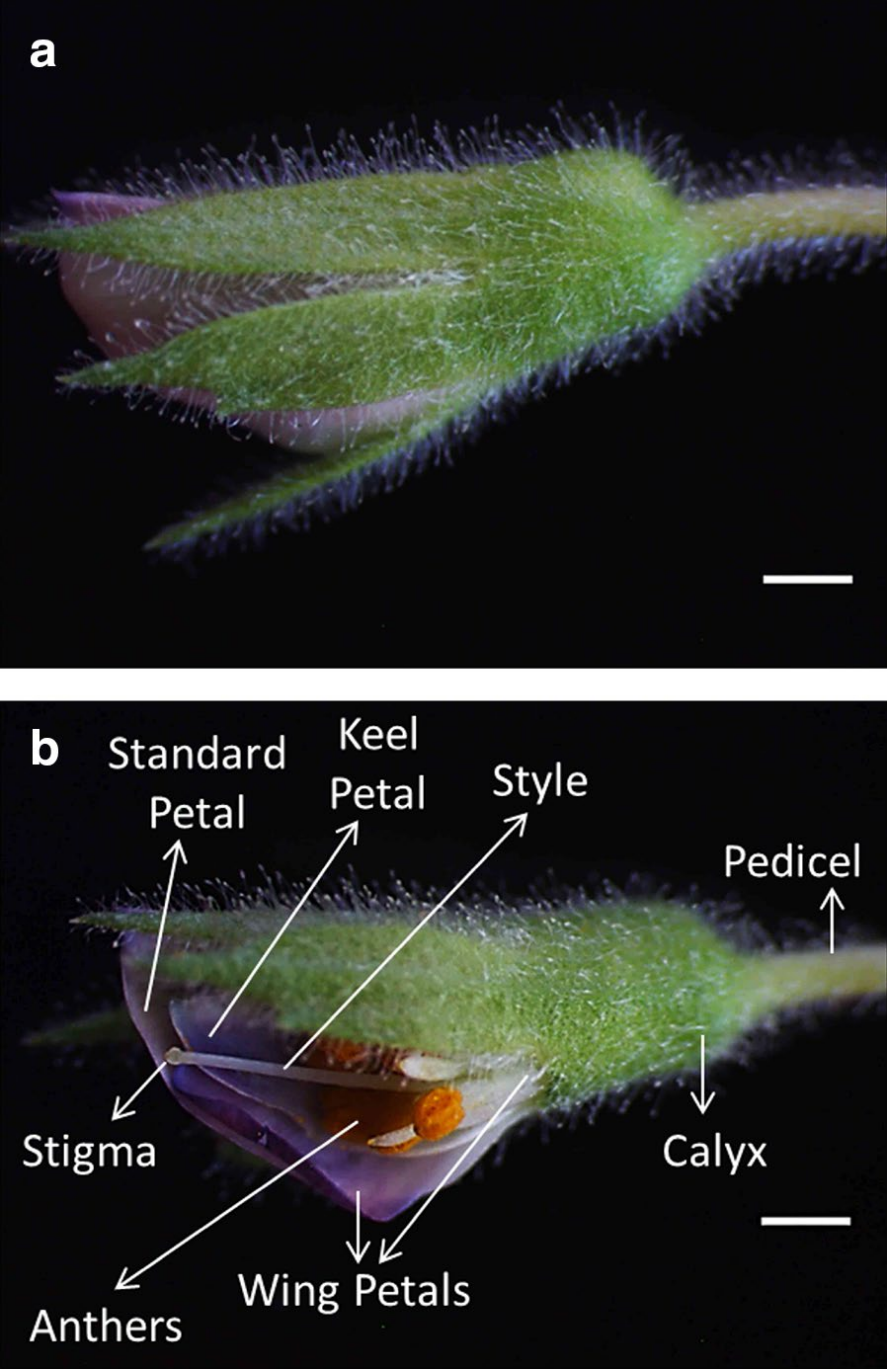
Flower structure of chickpea (*C. arietinum*). **a** Closed flower bud. **b** Different parts of flower bud. *Bars* 1 mm

Chickpea flowers are complete and bisexual and have typically papilionaceous corolla which contains five petals that include one large standard petal, two lateral wing petals and two fused keel petals which cover both male and female floral organs. The flower also comprised a calyx and pedicels. The stigma is globose and capitate and surrounded by anthers (Fig. 2).

**Fig. 3:**
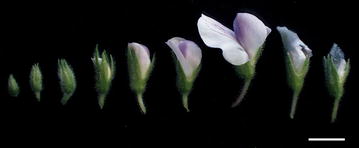
Various stages of flower development and senescence in chickpea. *Bar* 5 mm

**Fig. 4:**
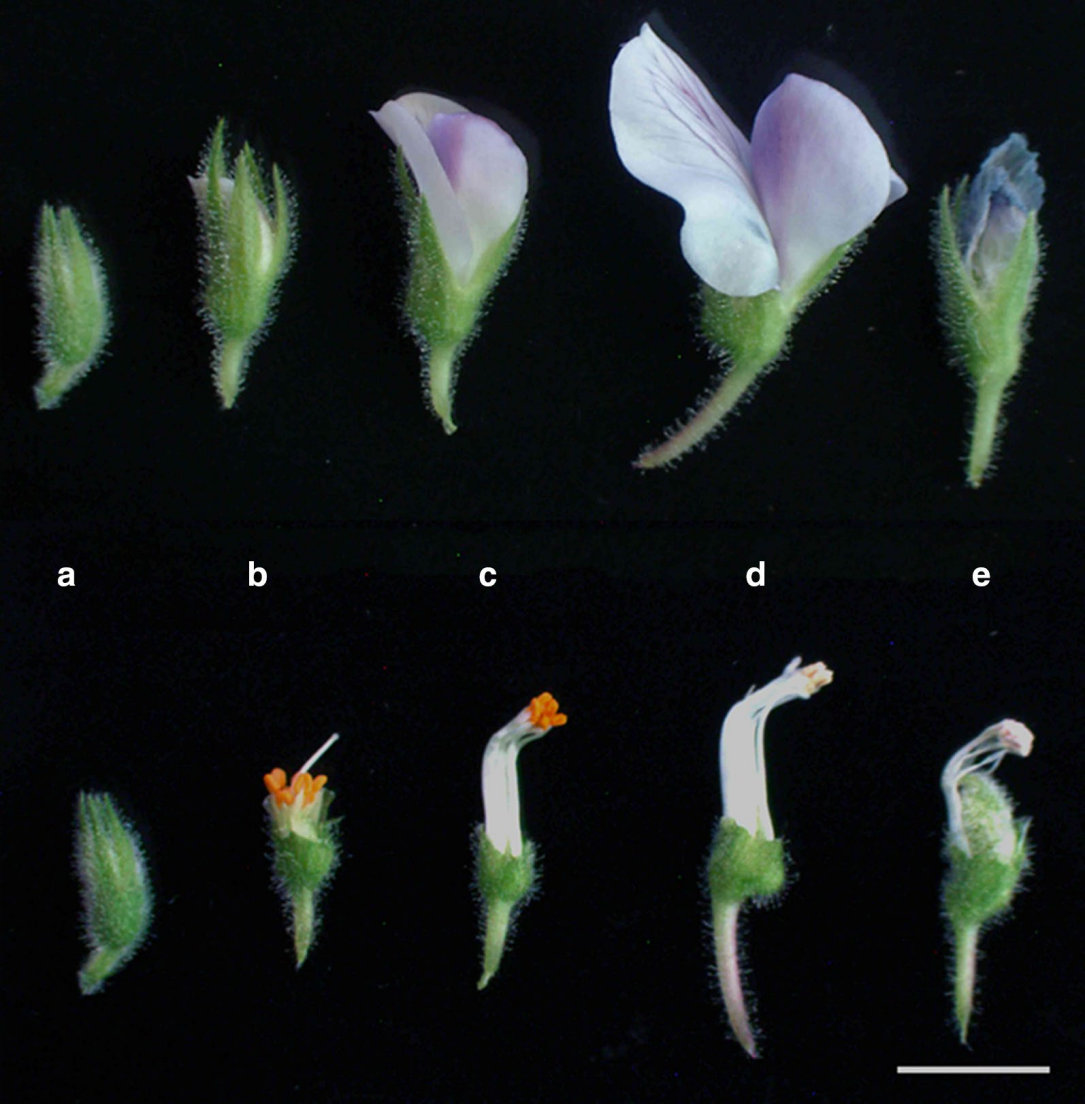
Five main stages of chickpea flower development. Petals and sepals are intact in *upper row* and detached in the *lower row*.** a** Closed bud stage where sepals cover petals in length at day 0.** b** Hooded bud stage when emasculation is done at day 1.** c** Half opened flower important for pollen collection at day 3.** d** Fully opened flower after self-pollination has occurred at day 4.** e** Faded flower where petals are wilted and ovary starts to expand at day 6. *Bar* 5 mm

Chickpea flower passes through various developmental stages during its growth (Fig. 3), and the following five important developmental stages of bud and flower were recognized by Eshel [15] (Fig. 4):

A. Closed bud: At this stage the stigma is immature and the anthers are still at the base of the bud (Fig. 4a).
B. Hooded bud: The corolla has elongated, and the anthers are about half the height of the style (Fig. 4b). The stigma is receptive and remains so until stage D.
C. Half open flower: At this stage anthers attain the same height as the stigma, and the pollen mature just before the dehiscence of anthers (Fig. 4c). Self-pollination takes place at this stage while the keel petal remains closed, preventing the entry of foreign pollen.
D. Fully open flower: The anthers become shriveled, while the standard and wing petals are fully expanded (Fig. 4d). Fertilization takes place 24 h after pollination [12].
E. Fading flower: This is the post-fertilization stage during which the ovary begins to elongate (Fig. 4e).

Female parent was grown until plants had one or two developing pods as this stage of development contains flower in all the different stages illustrated above (Fig. 3). For crossing, the hooded bud stage (Fig. 4b) was used for the emasculation in female parent as in this stage stigma is mature while anthers are not yet ripe. On the other hand, the male parent pollen for crossing was collected at half open flower stage (Fig. 4c) as this is the only stage where both stigma is receptive and pollen is mature for successful and efficient hybridization.

### Emasculation and artificial pollination

**Fig. 5:**
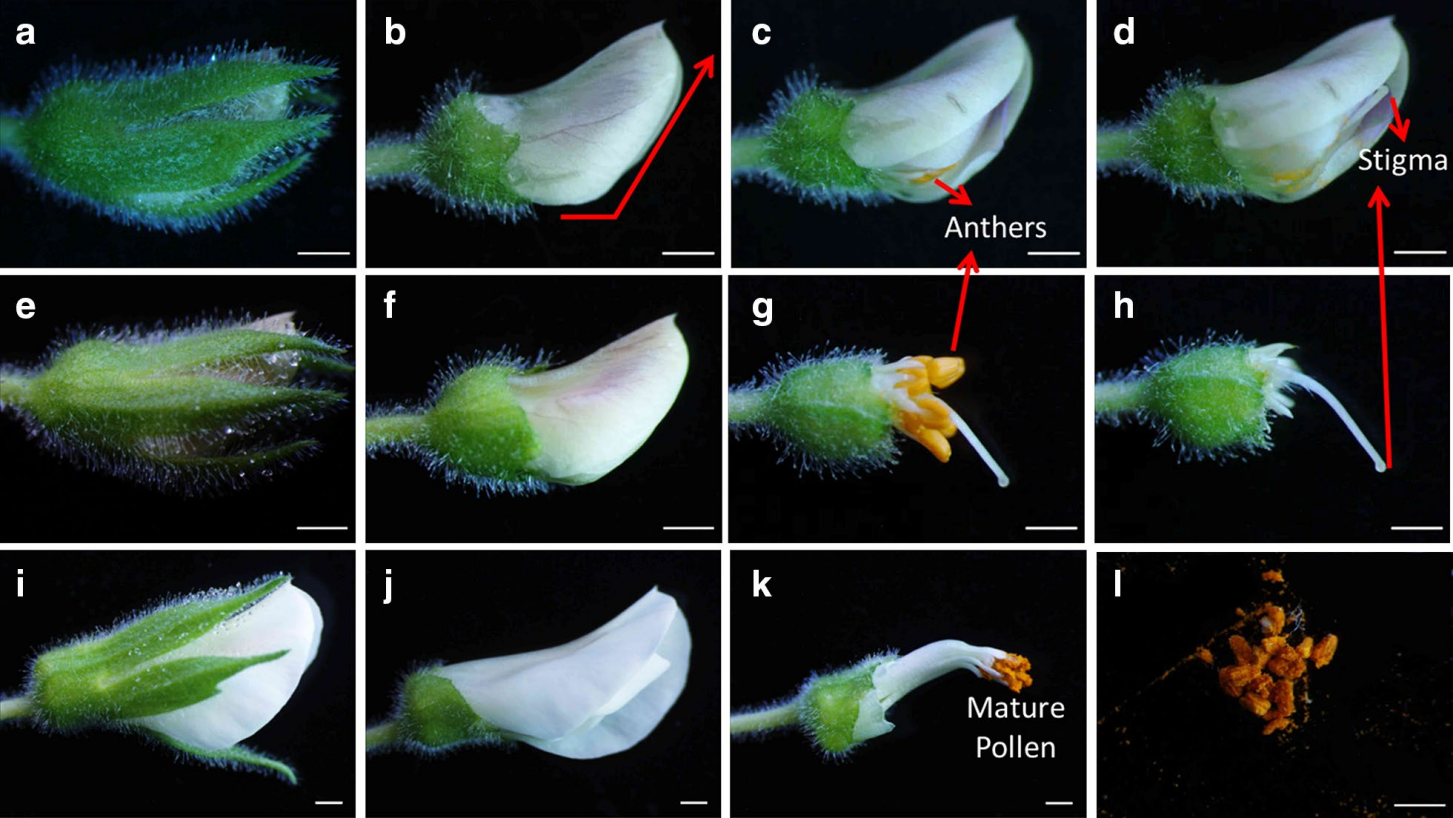
Parental preparation for crossing. Emasculation by keel petal incision method: **a** hooded flower bud chosen for emasculation of female flower. **b** Sepal removal for the keel petal incision. *Red line* shows the site of incision. **c** Flower bud showing the anther after keel petal incision. **d** Emasculated flower bud showing the stigma. Emasculation by petal removal method: **e** female flower bud selected for anther removal by petal excision method. **f** Detached sepals from bud. **g** Removal of standard, wing and keel petals exposing anther and stigma. **h** Emasculated female flower bud for cross-pollination. Pollen collection from male flower: **i** half open male flower for pollen collection. **j** Excision of sepals to expose the bud for petal removal. **k** Standard, wing and keel petal removal to collect the mature pollen. **l** Pollen collection on petriplate from 2 to 3 male flowers for cross-pollination. *Bars* 1 mm

After selecting a hooded bud (Fig. 5a) as female flower for emasculation, the front sepal was snipped off by sharp forceps (Fig. 5b). The keel petal was moderately cut at the bottom of the flower and incision was made along the central axis down to the distal end of the keel petal (Fig. 5c). This exposed the anthers (Fig. 5c), which were then removed from the filaments (Fig. 5d). Alternatively, if the temperature and humidity is controlled, there is no need to protect the pollinated flower by petals. Therefore, all the petals can be removed to expose the anthers for emasculation (Fig. 5e–h). Other internal organs such as peduncle, stigma and style are fragile and were not touched during emasculation.

**Fig. 6:**
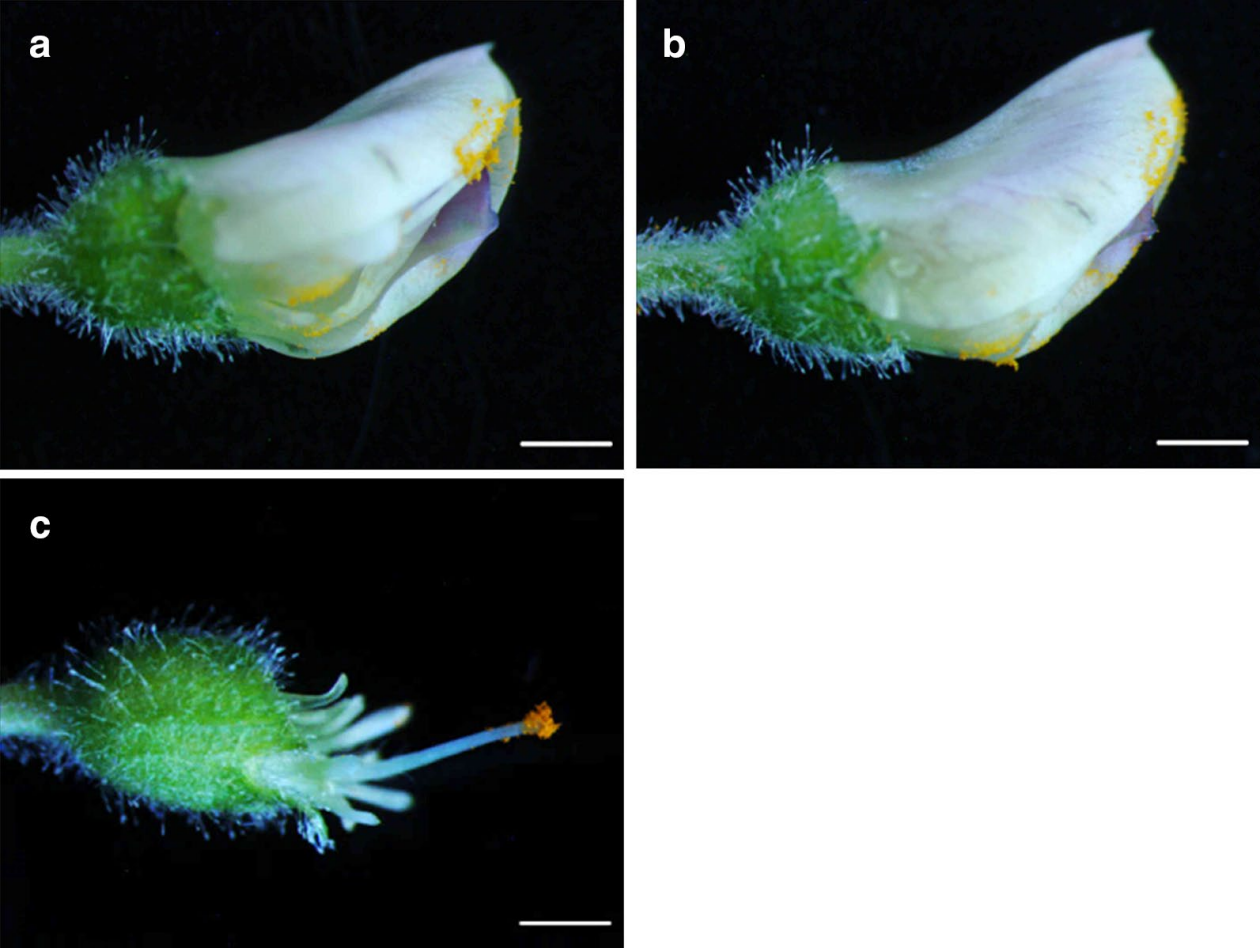
Process of artificial hybridization. **a** Placement of mature pollen on stigma exposed by keel petal incision method. **b** Closed flower bud after pollination. **c** Pollen covers the stigma which is uncovered by petal removal method. *Bars* 1 mm

Half open flowers were selected in male flower for pollen collection (Fig. 5i–j). Pollen at this stage is entirely mature, yellow in color and slightly sticky (Fig. 5k). To collect the pollen, all petals around the anthers were removed in the sequence where standard petal was removed first then wing and keel petals (Fig. 5k). This prevents the damage of anthers. Mature pollen grains were collected in petriplate (Fig. 5l) and were applied gently on the tip of the stigma of the emasculated female parent (Fig. 6a, c). More flowers can be used if the pollen from one flower is insufficient to dust the stigma. After pollination the style is inserted back gently inside the keel petal and covered by wing petals and standard petals that protect stigma and pollen from desiccation (Fig. 6b). Moreover, under controlled environment when the optimum temperature is between 22 and 26 °C there is no need to protect the stigma with petals (Fig. 6c). Emasculation followed by pollination was performed in the morning or in the evening when the outside temperature was cooler.

**Fig. 7:**
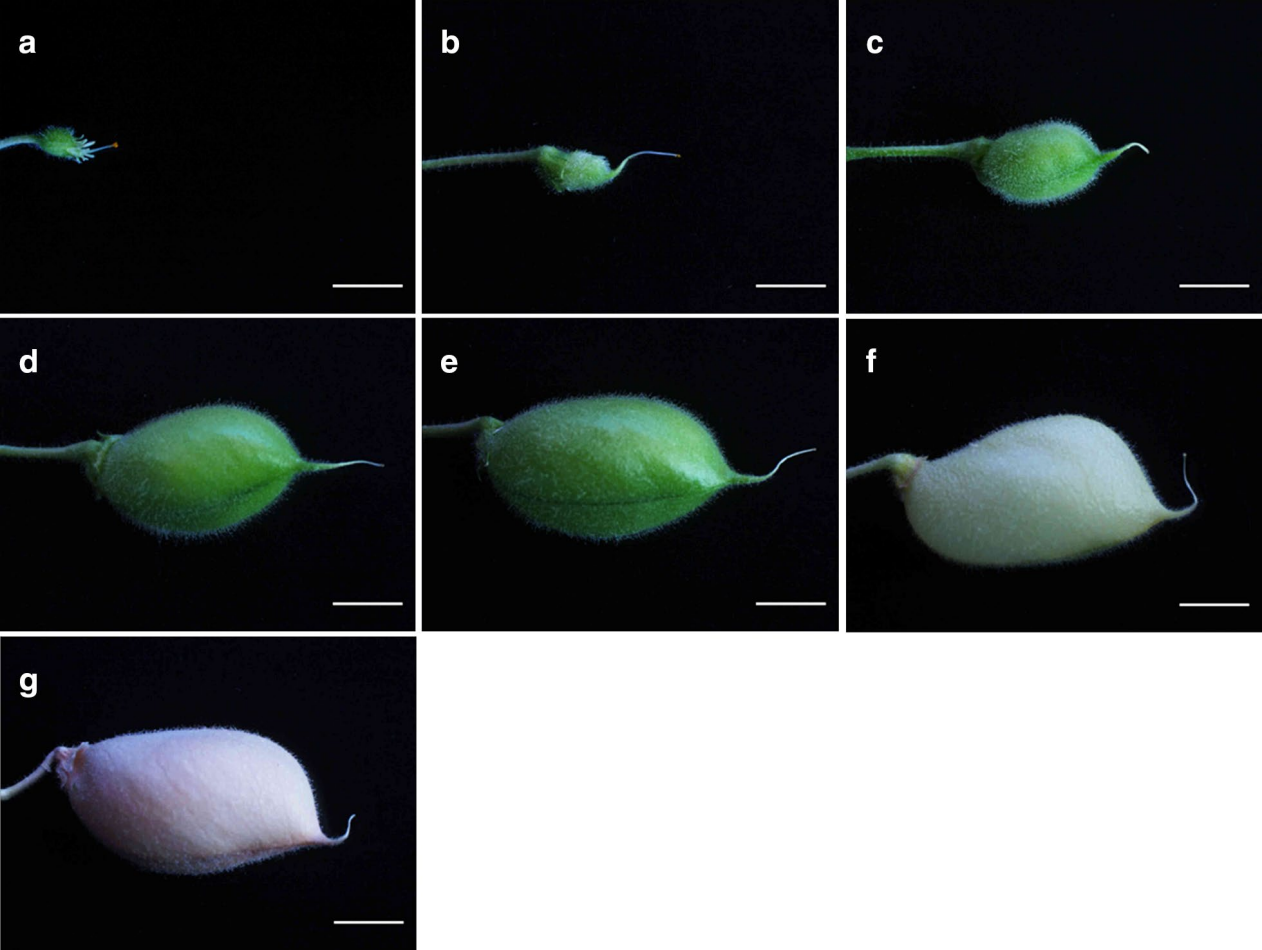
Pod development from successful cross-pollination. **a** Cross-pollinated female flower. **b** Pod initiation after 5 days of post fertilization. **c**–**f** Various stages of pod development. **g** Mature pod. *Bars* 5 mm

**Fig. 8:**
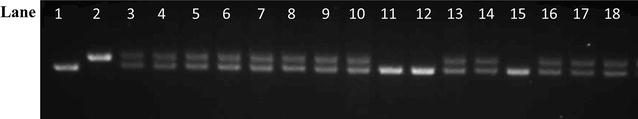
Hybridization testing through SSR marker. *Lane 1* parent 1 (ICCV96029), *lane 2* parent 2 (PI503023), *lane 3–17* F1 hybrid plants, *lane 18* positive control (mix of both the parental DNA). *Lane 3–10*, *13–14* and *16–17* represents successful crosses while *lane 11–12* and *15* shows unsuccessful crosses

The pollinated flowers were tagged and labeled. We kept track of artificially pollinated flowers (Fig. 7a) daily and trimmed new shoot or flower growing near to it. Pods from cross pollination were developed after 5 days (Fig. 7b) which went through different developmental stages (Fig. 7c–f) and were collected once matured and dried (Fig. 7g). F1 plants from collected seeds were grown under controlled conditions in greenhouse. Leaves of 1 week old plants were collected for DNA extraction and genotyping by PCR. Around 78% of the crosses showed the presence of both the parental DNA confirming successful hybridization and remaining 22% showed only the female parent genotype (Fig. 8) while around 33% of the crosses died before setting seeds (Table 1).

**Table 1:** Results of crossing success in chickpea

Crossing parents	Total number of buds crossed	Seed pods formed	Rate of seed pod set (%)	Pure hybrids	Crossing success rate (%)
ICCV96029 × 503023	160	108	67	84	78

## Conclusions

Artificial hybridization is an important process to develop genetically improved varieties of plants with desirable and novel characters from existing gene pools. In present study we have explained an easy and efficient method for genetic crossing in chickpea by keel petal incision or petal removal. We have described that the hooded bud is the most precise flower stage for emasculation while half-opened flower is for pollen collection, achieving approximately 78% success rate. Moreover, our study demonstrates that the success of hybridization can be influenced by the selection of parents and temperature. In conclusion, our detailed method for artificial hybridization can provide a guideline to enhance crossing efficiency in chickpea in order to create desired germplasm.

## Methods

### Plant material and growth conditions

*Cicer arietinum* ecotypes; desi (ICCV96029) and kabuli (PI503023) with wide variation in flowering time, leaf shape and seed size were grown in 1 gallon pots with BM7 soil (American Plant Products) and slow release Osmocote Classic fertilizer (American Plant Products) with NPK 14:14:14 in the green house under long day conditions at 22–30 °C in 16 h day and 8 h night regime. Plants were irrigated as required.

### Genetic crossing

Preparation of the female parent must coincide with the availability of the pollen from the male. Therefore, PI503023 was grown 3 weeks earlier as a late flowering male parent while ICCV96029 was grown later as an early flowering female parent. The crosses were performed in the greenhouse between 08:00 and 10:00 h in the morning and 17:00 and 18:00 h in the evening. For artificial hybridization, keel petal incision along the central line of flower bud was made by VWR^®^ fine tip forceps for emasculation and to expose the stigma for pollen deposition from male parent. Mature pollen grains were collected through fine tip forceps in a small petriplate and were placed on the stigma with the help of forceps or stigma can directly be dipped into collected pollen. Alternatively, pollen from male flower can directly be used for cross-pollination. Emasculation was always followed by pollination. After artificial pollination, the stigma with its deposited pollen was covered by keel petal, wing petals and standard petals to avoid drying of internal floral organs. Otherwise, if the temperature is between 22 and 26 °C, all the petals were removed for emasculation and stigma is covered by mature pollen and pollinated flower was left open for artificial hybridization (Additional file 1: Video S1). All the cross-pollinated flowers were tagged on the stem or flower stalk and labeled. After 5 days of cross-pollination flowers continued to develop pods. Pods were harvested once they became mature and dried; they were further dried for 1–2 days at 37 °C. Images of *C. arietinum* flowers and crossing techniques were obtained using Olympus SZX16 stereomicroscope.

### Genotyping of crosses

Seeds from dried pods were collected and F1 plants were grown in greenhouse under long day condition. 1–2 young leaves of 7 days old plants were collected for DNA extraction and genotyping by PCR. To extract the genomic DNA leaves were placed in an eppendorf tube and 400 µl of DNA extraction buffer [16] was added. Leaf tissue with extraction buffer was briefly ground with an autoclaved plastic pestle. Further, 60 µl of chloroform was added and samples were moderately mix by shaking for 2–5 min. Samples were then centrifuged for 10–15 min at 10,000–13,000 rpm. 300 µl of the upper phase was transferred in a new tube and 300 µl of isopropanol was added to it. Tubes were inverted several times and were centrifuged as above. Supernatant was discarded and pellet was washed with 500 µl of 70% ethanol. Then samples were centrifuged at 10,000 rpm for 5 min. Supernatant was removed and pellet was dried and resuspended in TE buffer (10 mM Tris and 1 mM EDTA, pH-8). DNA of the hybrid plants was screened by PCR for polymorphism using SSR markers that readily distinguish the parents [17]. Parental DNA template was used as positive control. The PCR temperature regime comprised an initial denaturation for 3 min at 94 °C, followed by 35 cycles of denaturation at 94 °C for 30 s, annealing for 30 s at 57 °C and elongation at 60 °C for 50 s and final elongation at 60 °C for 5 min. The PCR products were evaluated on 3% agarose gel stained with ethidium bromide. The SSR primer pairs, H4GO7_F, ATTAGAGGCAAACAAGAACTTGAAAC and H4GO7_R, TGACACCTAATTTTATTCGGTTTTTAT clearly showed the variability between two parents and were reproducible when used to characterize the F1 plants.

## Additional file

**Additional file 1: Video S1.** Method for genetic crossing in *C. arietinum*. The method is narrated step-by-step: https://youtu.be/ZTgDUcLGc_o.

## Abbreviations

DNA: deoxyribonucleic acid
PCR: polymerase chain reaction
*C. arietinum*: *Cicer arietinum*
h: hours
min: minutes
sec: seconds
